# Alteration of ankle proprioceptive threshold during gait in the presence of acute experimental pain

**DOI:** 10.1371/journal.pone.0263161

**Published:** 2022-01-25

**Authors:** Michaël Bertrand-Charette, Miorie Le Quang, Jean-Sébastien Roy, Laurent J. Bouyer

**Affiliations:** 1 Center for Interdisciplinary Research in Rehabilitation and Social Integration (CIRRIS), Quebec City, Quebec, Canada; 2 Department of Rehabilitation, Faculty of Medicine, Laval University, Quebec City, Quebec, Canada; University of Illinois at Urbana-Champaign, UNITED STATES

## Abstract

**Objective:**

Human gait requires complex somatosensory processing of various inputs such as proprioception. Proprioception can be altered in the presence of pain. This has been shown mostly during controlled tasks, thereby limiting the influence of external perturbations. While controlling the environment is sometimes warranted, it limits the ecological validity of the data. Using robotic orthoses to apply perturbations during movements seems a promising tool to functionally assess proprioception, where the complex somatosensory processing required in real-life situations is at play. The main objective of this study was to compare the proprioceptive threshold of healthy participants during gait in the presence and absence of an acute experimental pain.

**Methods:**

36 healthy participants walked on a treadmill while wearing a robotized ankle–foot orthosis (rAFO) around their right ankle. The rAFO applied torque perturbations of graded magnitudes during the swing phase of gait. Participants had to report the presence/absence of such perturbations, as a measure of proprioceptive threshold. Following initial assessment, they were randomly assigned to one of three experimental groups: Control (no stimulation), Painless (non-nociceptive stimulation) and Painful (nociceptive stimulation). Electrodes placed on the right lateral malleolus delivered an electrical stimulation during the second assessment for Painless and Painful groups. A Kruskal-Wallis was used to compare the percentage of change of the three groups between the two assessments.

**Results:**

A 31.80±32.94% increase in proprioceptive threshold, representing an increase of 1.3±1.2 Nm in the detection threshold, was observed for the Painful group only (p<0.005), with an effect size of 1.6.

**Conclusion:**

Findings show that the presence of pain at the ankle can alter participants’ proprioceptive threshold during gait. Clinical assessment of proprioception should therefore carefully consider the presence of pain when evaluating a patient’s performance using clinical proprioceptive test and consider the negative effect of pain on proprioceptive threshold for test interpretation.

## Introduction

Human gait is a relatively simple task requiring complex somatosensory processing [1]. To walk successfully under different environmental conditions, the central nervous system has to process information originating from various stimuli including visual [2], cutaneous [3] and proprioceptive [4] to adapt gait and avoid injuries.

Proprioception can be defined as a group of senses that inform the central nervous system on body segments position and movement (kinesthesia), muscle tension/force, effort and balance [5, 6]. It is an important contributor to dynamic control of joint stability [7]. It uses sensory information from sensors located in several structures of the body including the skin over joints, muscles, tendons, fascia, joint capsules and ligaments [5, 8].

The proprioceptive senses can be altered by several factors [7] such as injury and pain. Indeed, musculoskeletal injuries, such as lateral ankle sprains (LAS), can cause direct trauma to the mechanoreceptors in ligaments leading to a decrease in limb position and movement sensing [9–13]. Indirect alteration of proprioception can also arise from intact receptors providing incorrect information in the presence of swelling or hemarthrosis [7]. Regarding pain, Lee et al. (2010) have reported that people suffering from low back pain have significantly higher movement perception threshold than healthy controls [14]. Ager et al. (2020) have also shown that kinesthesia and the sense of force can be altered in the presence of shoulder pain [15]. Evidence of pain altering proprioception has been assessed at various body locations, such as the lower back [14], knee [16], neck [17] and shoulder [15]. Testing was usually performed during controlled tasks, thereby limiting the influence of external perturbations and distractors. While controlling the environment is sometimes warranted, it limits the ecological validity of the data obtained. Measuring the impact of pain on proprioception during a functional task, where the complex somatosensory processing required in real-life situations is at play, remains to be done.

One promising approach to assess proprioception during a functional task is presented in Fournier Belley et al. [18]. They used a robotized ankle-foot orthosis (rAFO) to test the ability of participants to detect a perturbation of graded magnitude presented at the ankle joint during walking. With this rAFO, they were able to assess proprioception in a reliable manner during gait in healthy participants.

The test described in Fournier Belley et al. assessed the proprioceptive threshold, i.e. the minimal movement error detectable by each participant during actual movement execution. Using the same rAFO while inducing experimentally controlled pain, would therefore make it possible to assess the impact of pain on proprioception during a functional task.

The objective of this study was therefore to compare the proprioceptive thresholds of healthy participants in the presence and absence of acute experimental pain during gait. Participants were divided into three groups (no pain, non-nociceptive electrical stimulation, and nociceptive electrical stimulation) and completed the proprioceptive threshold test twice. The first test was performed without pain, while the second depended on the assigned group. Based on previous studies assessing proprioception in controlled tasks, it was hypothesized that pain would also interfere with proprioception during gait, leading to an increased movement error detection threshold for the painful group. Furthermore, if this effect is specific to pain (and not simply due to the distraction induced by the stimulation protocol), the subgroup receiving non-painful stimulation should show no change in their movement error detection threshold across the 2 tests.

## Methods

### Participants

A convenience sample of 36 young healthy adults (27.3 ± 4.1 years old; 20 females) was recruited from the *Université Laval* student population. The sample size was calculated from Fournier Belley et al. [18]. Participants were naïve to the task and research hypotheses, free from lower limb injuries (last 6 months; self-reported), and had no known neurological impairment that could affect task performance. They were excluded if they reported pain or other conditions that could affect task performance prior to testing onset. All participants read and signed a consent form describing the experimental procedure and their involvement in the study. This protocol was approved by the local ethics review board (CIUSSS-CN, #2010–212). The experimental procedures were in accordance with the Declaration of Helsinki.

### General protocol

**Fig 1:**
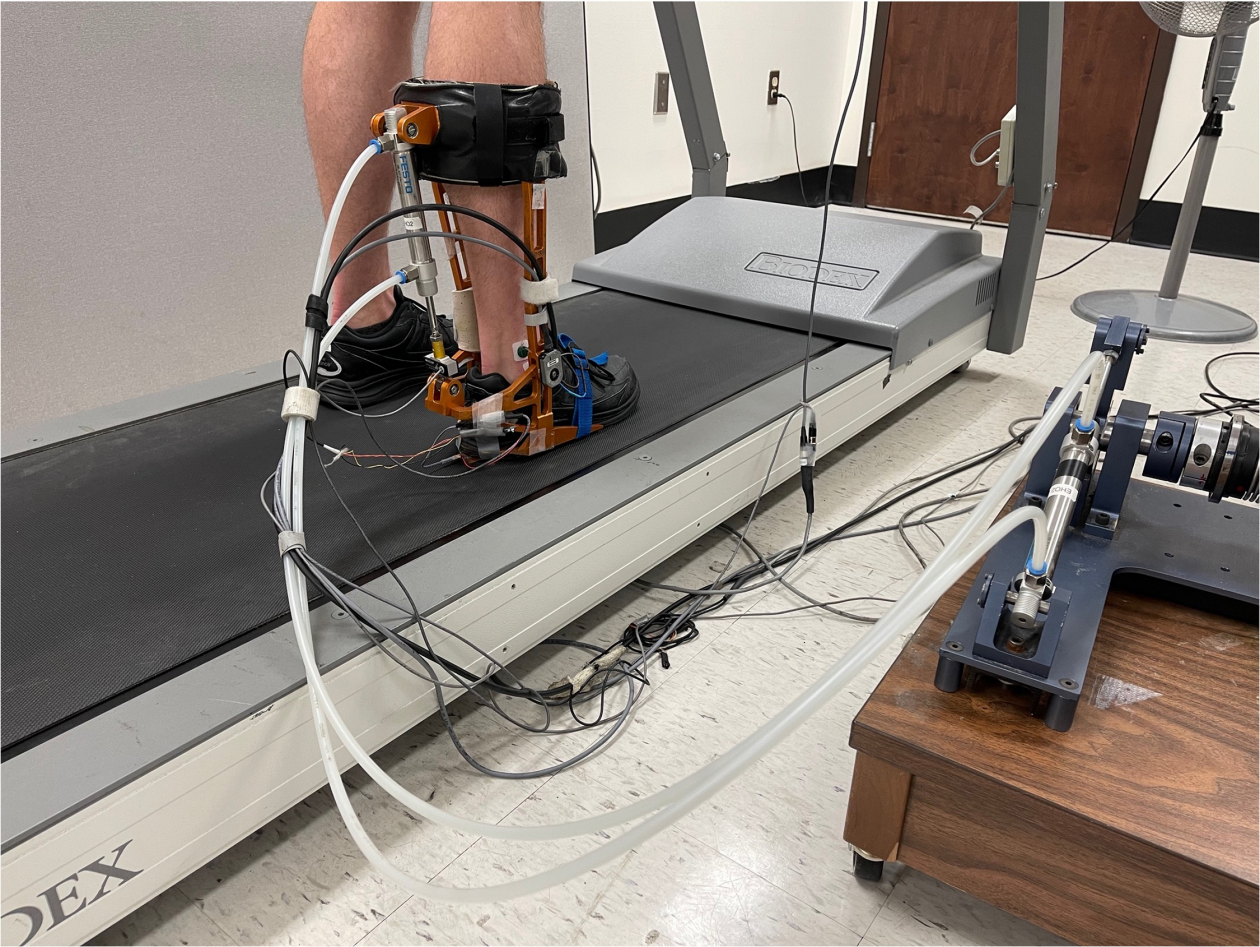
Electrohydraulic robotized ankle–foot orthosis (rAFO).

Participants came to the laboratory for a single visit. They first completed the Lower Extremity Functional Scale (LEFS; a self-reported questionnaire used to assess lower limb function [19]) and the Waterloo Footedness Questionnaire (WFQ) to assess foot preference [20]. Their sociodemographic characteristics were then collected. Upon completion of the questionnaires, participants walked on a treadmill at 3.6 km/h while wearing the robotized ankle–foot orthosis (rAFO) on their right ankle (Fig 1). They were then accustomed to walking with the rAFO and to feel the different movement perturbation magnitudes during a 5-minute familiarization period.

Thereafter, they performed a first proprioceptive threshold assessment test to determine their individual baseline value (see description below). This was followed by a 15-minute break during which they rested and were randomized to one of the three following groups: (1) *Control group*, where the second proprioceptive threshold assessment test was performed without any electrical stimulation; (2) *Painless stimulation group*, in which participants received non-nociceptive electrical stimulation at the ankle during the second proprioceptive threshold assessment test; and (3) *Painful stimulation group*, in which participants received nociceptive electrical stimulation at the ankle during the second proprioceptive threshold assessment test.

During the break, participants in the Painless and Painful groups were familiarized with electrical stimulation. Stimulation electrodes were placed on the right lateral malleolus and at the distal end of the fibula of the right limb. Intensity of the electrical stimulation was then adjusted to either Painless (1.2x stimulation perception threshold (PT)) or Painful (4/10 on a numeric VAS, assessed during gait). Stimulation onset was triggered by a foot switch located under the right heel and was only present during right heel contact with the ground (see Bertrand-Charette et al. (2021) for further details [21]).

### Ankle torque perturbation test to assess proprioceptive threshold

**Fig 2:**
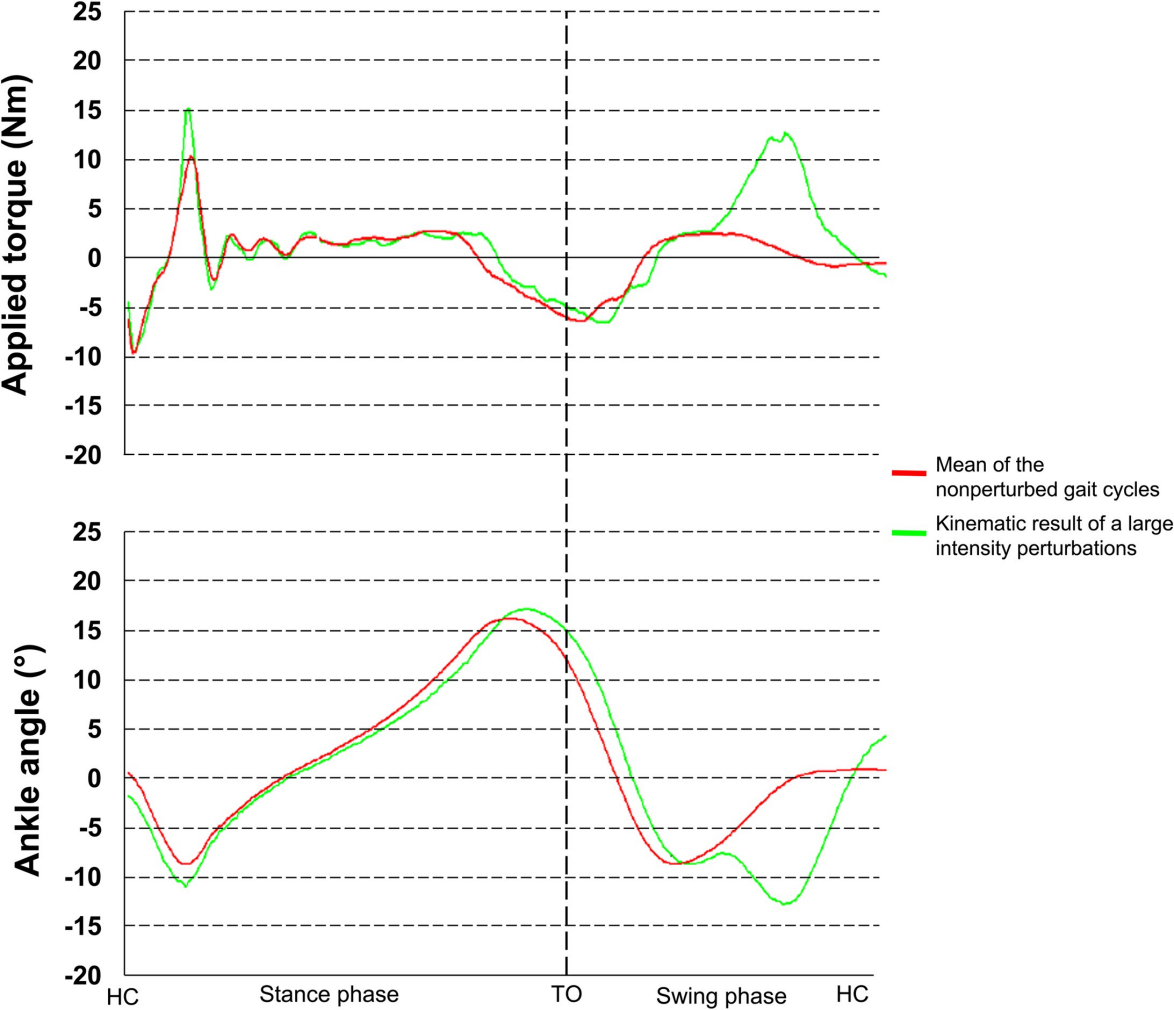
Deviations produced by the torque perturbation. The mean of the nonperturbed gait cycles is represented by the red line, while the kinematic result to a simple torque perturbation is shown in green for the applied torque and the ankle angle.

A custom-designed rAFO [22] was used to apply torque perturbations towards plantarflexion during the mid-swing phase of the right lower limb (Fig 2). The amplitude of the perturbation was adjustable, and produced corresponding small to large ankle angular deviation towards plantarflexion, modifying the normal trajectory of the ankle to study ankle proprioception, as previously described by Fournier Belley et al. (2016) [18] and Dambreville et al. (2019) [23]. The perturbation was randomized every 3rd to 7th stride to prevent anticipation from the participant. The exact timing of the perturbation was tailored to each participant’s gait pattern (see Fournier Belley et al. [18] for more details). Briefly, the baseline gait pattern of each participant was recorded during the familiarization period. The torque perturbation timing was adjusted such that the deviation occurred during dorsiflexion.

Perturbation magnitude ranged from 0.5 to 20 Nm and was adjusted during the experiment to determine the proprioceptive threshold. Participants were asked to “walk normally while looking straight ahead” and to press a push button whenever they felt a perturbation while walking. To adjust the magnitude of the perturbation throughout the experiment, a modified version of the Parameter Estimation by Sequential Testing (PEST) method was used, as described in Choi et al. (2016) [3]. Briefly, after the first perturbation, the PEST method determined the next magnitude based on the participants’ response: It was either reduced or increased, depending if the participant detected or not the perturbation, respectively. To reduce biased estimation, participants had to identify correctly 2 out of 3 times the same perturbation before the torque magnitude was changed by the PEST algorithm (see [23]).

### Electrical stimulation

Trains of 5 pulses at 300 Hz (pulse width 500μs) were generated by two electrical stimulators (s-88, Grass Instruments, Quincy, MA, USA). These parameters were selected based on a previous study in order to generate a musculoskeletal-like pain that is phase-specific and local at the ankle [21]. The stimulation was delivered through a Digitimer DS7A stimulator (Hertfordshire, United Kingdom). Two electrodes were placed 2 cm apart longitudinally over the right lateral malleolus and fibula. Care was taken to avoid radiating pain and producing only a local stimulation around the lateral malleolus by adjusting electrode placement. For the Painless group, increases in steps of 5 mA were used to individually adjust the stimulus intensity until the PT was reached. Final stimulus intensity was set at 1.2 x PT. For the Painful group, the same increases in steps of 5 mA were used until a pain level of 4/10 on a numeric VAS was reached. Intensity of stimulation was adjusted during the second half of testing if needed, to ensure that a 4/10 pain level remained throughout the experiment.

### Recordings and data analysis

**Fig 3:**
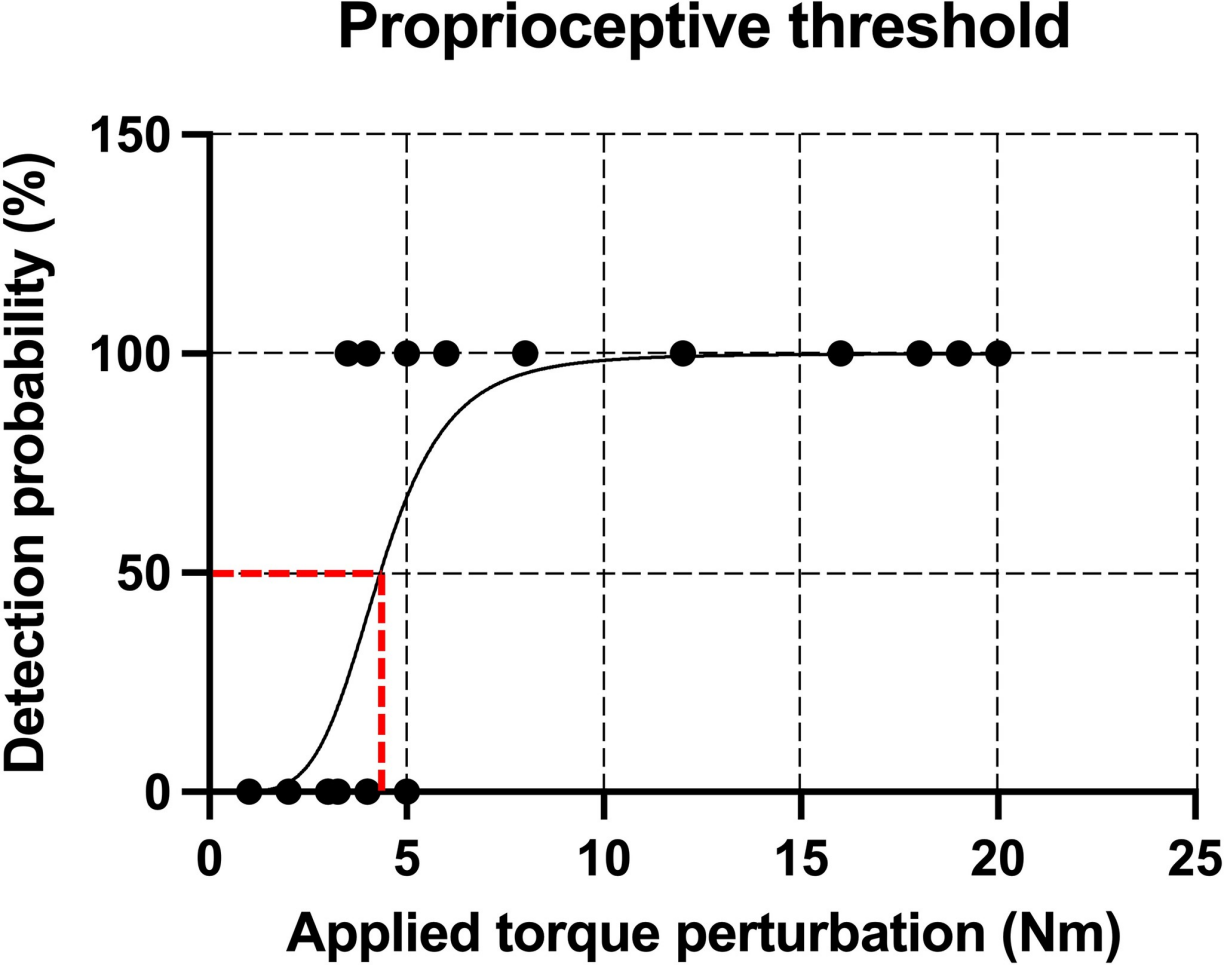
Determination of the proprioceptive threshold. A sigmoidal curve (black line) was fitted to the data, and the torque perturbation detection threshold (Nm) was determined as the 50% detection level (red dashed line).

Each perturbation identification attempt (successful or not) was recorded manually by one member of the experimental team. Following the experiment, all attempts were transferred to a spreadsheet. Then, a score of 100% was given to successful detections for each perturbation intensity, and a score of 0% to unsuccessful ones. A plot of responses to the perturbation (100% = detected, 0% = not detected) as a function of perturbation intensity (applied torque; Nm) was then created for each participant using GraphPad Prism 9.2.0 (San Diego, CA) (see Fig 3). A sigmoidal curve was then fitted to the data, and the proprioceptive threshold (Nm) was determined as the 50% detection level (see Fournier Belley et al. [18] for more details). The thresholds from the first and second tests were then compared, and a percentage of change was determined relative to baseline (100*(Threshold 2-Threshold 1) /Threshold 1).

### Statistics

As the data was not normally distributed, a Kruskal-Wallis test was used to compare the percentage of change between the three experimental groups using GraphPad Prism 9.2.0. An effect size was calculated using the following formula: (mean of Painful group–mean of Control group)/standard deviation of Control group). Dunn’s multiple comparisons test was performed for post-hoc analysis when a significant Group effect was observed. For post-hoc analysis, the mean of the painful and painless groups was only compared to the mean of the Control group to limit type I error. Significance level for both groups and intra-subject analysis was set at 0.05.

## Results

### Participants’ characteristics

The group was composed of 36 healthy participants aged between 19 and 36 years old (mean of 27.3 ± 4.1; 20 females; see Table 1). Participants were then randomized in one of the three experimental group (n = 12 per group).

**Table 1:** Participants’ characteristics.

Characteristic	Control	Painless	Painful	p
n	12	12	12	n.a.
Age	28.3±2.3	26.4±3.8	27.0±5.7	p = 0.27
Sex	5 M; 7F	5 M; 7F	6 M; 6F	p = 0.90
Footedness	10 R; 2 L	11 R; 1 L	9 R; 3 L	p = 0.56
Height (cm)	172.6±9.4	173.4±7.9	172.3±9.4	p = 0.95
Weight (kg)	73.7±11.2	71.3±16.0	68.3±10.8	p = 0.80
Perturbation Timing (% of gait cycle)	69.3±2.4	69.3±2.6	67.8±2.9	p = 0.26
Stimulation intensity (mA)	0	2.4±1.4	8.6±2.7	p<0.0001
VAS score (/10)	0	0	4	n.a.

cm = Centimeters; kg = Kilograms; F = Female; L = Left; mA = Milliamps; M = Male; R = Right; n.a. = Not applicable; VAS = Visual Analog Scale.

### Stimulus intensity during the proprioceptive threshold assessment test

During the second proprioceptive threshold assessment test, the mean intensity of the electrical stimulation delivered was 2.4±1.4 mA for participants in the Painless Group and 8.6±2.7 mA for participants in the Painful group, resulting in a mean VAS score of 0 and 4, respectively. Stimulus intensity was significantly higher in the Painful group (p<0.0001).

### Effect of pain on proprioceptive threshold

**Fig 4:**
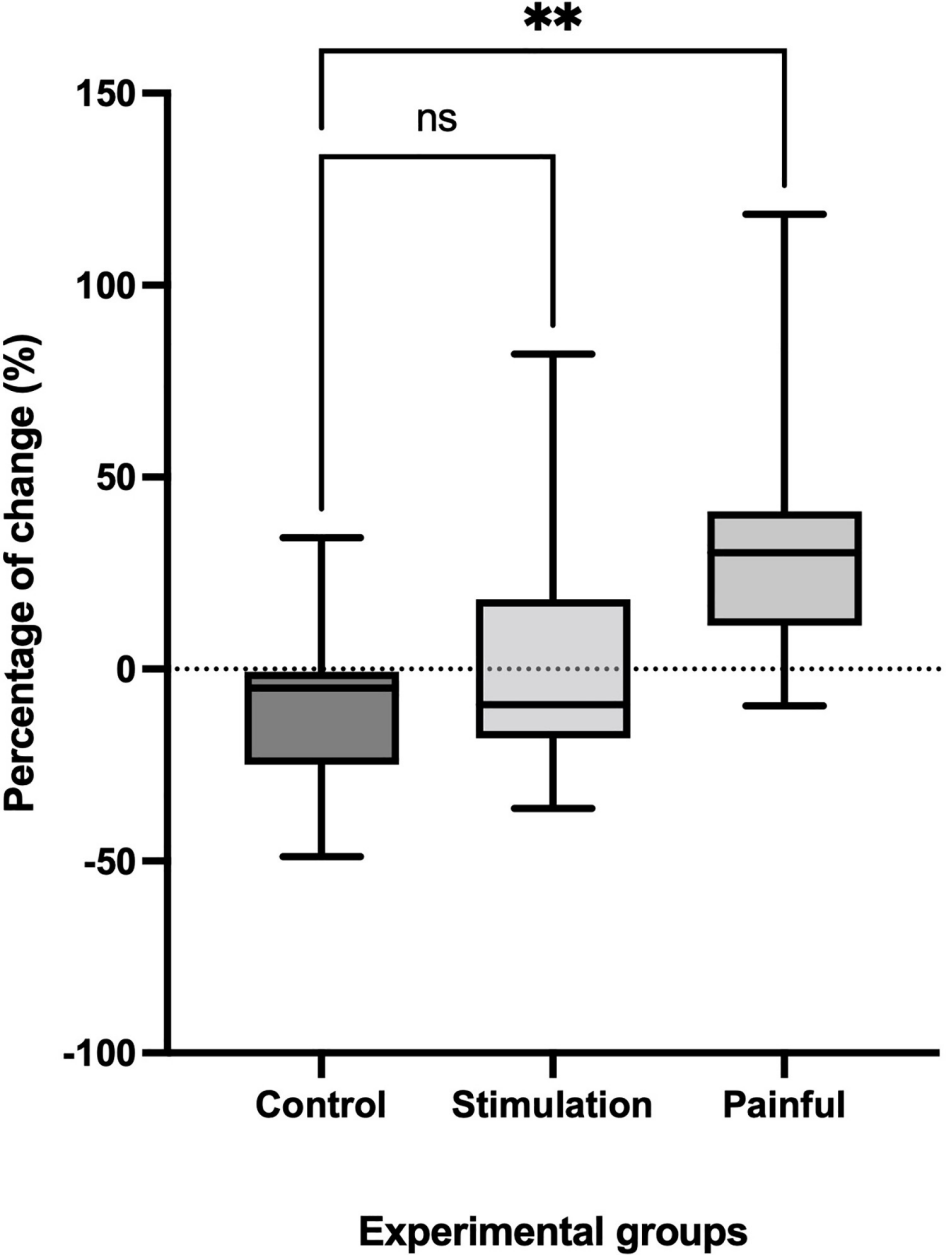
Bar graph for Kruskal-Wallis one-way analysis of variance. Result of the non-parametric analysis of variance comparing the three groups. ** = p<0.01; ns = not significant.

The percentage of change for the second proprioceptive threshold assessment test with respect to baseline is presented in Fig 4. There was a significant Group effect (p<0.01). Post hoc Dunn’s multiple comparison test showed no difference (p>0.99) between the Control and Painless groups (mean rank difference: -0.33%). On the other hand, a statistically significant difference was observed between Control and Painful (p<0.01), with a mean rank difference of -12.42%. Overall, the Painful group showed a mean significant increase in proprioceptive threshold of 31.80±32.94% (p<0.005), with an effect size of 1.6. This change represents an increase of 1.3±1.2 Nm in the detection threshold in the presence of acute pain.

## Discussion

The present study demonstrates that the presence of an experimentally controlled nociceptive stimulation at the ankle during gait can alter ankle proprioceptive threshold. Furthermore, as this effect was not present in the Painless group, our results suggest that the proprioceptive threshold increase was not simply due to distraction by the electrical stimulation, but was specific to pain.

### Effect of pain on ankle proprioception

During gait, participants in the Painful group showed an increase of 31.80±32.94% in their ankle proprioception threshold, with an effect size of 1.6. Their proprioceptive acuity at noticing movement errors during gait therefore decreased. These results confirm our hypothesis and are similar to previous studies assessing the influence of pain on proprioception at various body sites during controlled tasks. As mentioned in the introduction, Ager et al. reported that kinesthesia and the sense of force at the shoulder are altered in the presence of pain [15], and Lee et al. showed that LBP patients had a significantly greater motion perception threshold than controls, while showing no difference in a repositioning task [14]. In addition, in a study on Joint Position Sense (JPS) at the knee that compared individuals with patellofemoral pain syndrome to healthy controls, altered JPS was reported in the affected knee, together with a significant loss in proprioception of the contralateral normal knee [16]. Together these studies support the results of the current work, where only the Painful group showed a statistically significant proprioceptive deficit in the presence of pain. The novelty is that our results were obtained in a functional task, where the full somatosensory processing of “real-life” is present, including sensory gating. The latter could partly explain the large effect size of our results. Additionally, the 1.6 effect size is larger than the ones obtained by Lee et al. [14], in which effect sizes for motion perception threshold ranged from 0.4 (trunk axial rotation) to 1.5 (trunk flexion).

### Ecological validity of testing

Using ankle proprioceptive threshold assessment test to assess proprioceptive threshold has been shown to be a reliable way to study proprioception during gait [18]. It seems reasonable to state that this test has a higher ecological validity by assessing global somatosensory processing instead of solely focusing on joint position sense during a repositioning task.

Moreover, as reported by Fournier Belley et al. [18], the movement error detection threshold is higher during the rAFO testing than what has been reported when the ankle was assessed in a static position (proprioceptive threshold of approximatively 5° compared to 0.5° - 2.5°) [18]. This could be explained by the fact that optimal human gait results from complex interactions between central drive, central pattern generators and somatosensory inputs [1, 24]. Furthermore, movement control itself involves an increase in the gating of sensory information [25]. The result of these complex interactions is likely associated with the increase in proprioceptive threshold observed during gait when compared to a static evaluation [25]. These findings therefore strongly support that proprioception should be assessed during functional tasks in addition to passive and active JPS testing. This has been highlighted in a recent systematic review [26].

### Interaction between pain and proprioception

As mentioned above, human gait requires complex somatosensory processing in order to maintain a stable locomotor pattern in the face of a wide range of externally imposed conditions [1]. As pain pathways project to the somatosensory cortex, the limbic system and the frontal cortex [27], pain could alter proprioception at several levels of the central nervous system and possibly be detrimental to performance during a functional task. Moreover, Frot et al. provided direct evidence for a spinothalamic input to the motor cortex in humans [28], further supporting the hypothesis of somatosensory gating of proprioceptive inputs. These findings highlight a possible detrimental interaction between proprioception and pain that could explain the decrease in proprioceptive threshold in the Painful group.

Regarding the more ‘clinical’ evidence, previous studies assessing proprioception using the Joint Position Sense (JPS) test mostly compared healthy participants to chronic ankle sprains or did not document the presence of pain [11, 12, 29–31]. It is therefore difficult to gain insight on the interactions between pain and proprioception on the sole basis of these studies. However, their results show that participants with sprained ankles had significant proprioceptive deficits during JPS testing compared to healthy participants. This can partly be explained by the injured anatomical structures that become less efficient for mechanical joint stabilization [7]. In the present study, ankle ligaments were intact. Therefore, the reduction in proprioceptive threshold can only be explained by the painful electrical stimulation. It is possible that competing sensory information interfered with the proprioception sense as mentioned above. It is also possible that the proprioceptive deficit observed in the Painful group is secondary to sensory gating taking place within the sensorimotor system. As stated in Fournier Belley et al., interactions between central drive, sensory feedback, and the musculoskeletal system are more complex during dynamic motor tasks [18]. These two theories could explain alone or in combination the results obtained in the present study.

### Clinical implications

It is well known that ankle sprains are associated with proprioceptive deficits [32–34]. Because of the high prevalence of ankle sprains [35] and its high chances of recurrence (as high as 73% [35, 36]), adequate assessment of proprioception following an ankle sprain is key to optimal treatment. Now knowing the possible detrimental interaction between pain and proprioception detection threshold, we recommend clinicians to systematically document the presence of pain during their clinical evaluation. Moreover, they should consider the presence of pain during functional evaluations, as it can significantly reduce the patient’s proprioception. The presence or absence of pain might influence the test’s results and should therefore be considered when interpretating a proprioceptive test result.

### Strengths and limitations of the study

There are some limitations to this study. First, participants in all three groups were relatively young adult, which might limit the generalizability of the results. Another limitation is the weight of the orthosis (1.7 kg), that might have slightly interfered with some participants normal gait pattern. However, a previous study showed that the rAFO impact on gait parameters are minimal [37].

This study also has several strengths. It highlights the fact that a painful stimulation can alter ankle proprioceptive threshold. Furthermore, the inclusion of a Painless group in the study design allowed us to conclude that the reported effect is specific to pain, and not simply a response to a focal cutaneous stimulation or to attention distraction. It is also the first study to look at the effect of acute experimental pain on ankle proprioception while walking. This functional test has higher ecological validity than static JPS testing. Finally, the use of an electrical nociceptive stimulation that creates a focused, acute, repeatable, and easily adjustable pain level made it possible to control pain intensity across participants in the Painful group.

### Conclusion

The presence of pain while assessing proprioception during gait has a negative impact on the ankle joint proprioceptive threshold. Our results highlight this alteration of proprioception in participants undergoing local acute pain, compared to participants without any pain. Clinically, this finding suggests that during the assessment of proprioception, pain should be documented to ensure accurate interpretation of the results, as pain has the potential to increase the proprioceptive threshold.

## Supporting information

S1 Data (XLSX)
